# Heterogeneous Presentations of iMCD: A Single-Institution Case Series

**DOI:** 10.1155/crh/3377688

**Published:** 2025-11-03

**Authors:** Jaspreet Kaur, Anthony Sisk, Jonathan E. Zuckerman, Haifaa Abdulhaq

**Affiliations:** ^1^Division of Hematology/Oncology, University of California San Francisco, Fresno 93701-2302, California, USA; ^2^Department of Pathology and Laboratory Medicine, David Geffen School of Medicine at UCLA, Los Angeles 90095, California, USA

**Keywords:** diagnosis, idiopathic multicentric Castleman disease, siltuximab, TAFRO

## Abstract

**Background:**

Idiopathic multicentric Castleman disease (iMCD) is a rare lymphadenopathic disorder characterized by hyperplasia of multiple lymph nodes and can be associated with a wide range of symptoms and presentations, from mild disease to life-threatening organ failure. Varied histopathological features and heterogeneous presentation of this rare entity can make the diagnosis quite challenging for both hematologists and other specialists who may encounter patients at various stages of disease progression.

**Method:**

We analyze five different clinical presentations at our institution to demonstrate challenging routes of diagnosis and treatment complexities of iMCD. We aim to raise awareness to the importance of early diagnosis and appropriate management of this rare condition.

**Results:**

All patients in this series presented with symptomatic lymphadenopathy. We highlight one rare instance of thrombocytopenia, anasarca/ascites, fever, reticulin fibrosis or renal dysfunction, and organomegaly (TAFRO) syndrome with elusive iMCD, which illustrates the challenges in the diagnosis of this rare condition and the importance of early recognition of its symptoms to avoid decompensation of patients. We also review established treatment guidelines and response criteria to siltuximab as outlined in the international consensus treatment guidelines.

**Conclusion:**

These cases highlight the heterogeneity and challenging diagnosis of this rare cytokine-driven hematological disorder and the role of siltuximab in the treatment of iMCD.

## 1. Introduction

First identified in the 1950s, Castleman disease (CD) continues to be a challenging hematologic disorder due to its rare occurrence and wide variety of symptoms. The disease can be classified as unicentric CD (UCD, with involvement of a single lymph node station) and multicentric CD (MCD, with ≥ 2 lymph node stations involved) [1]. MCD can be further divided into human herpes virus type 8 (HHV-8)–associated MCD, polyneuropathy, organomegaly, endocrinopathy, monoclonal plasma cell disorder, skin changes (POEMS)–associated MCD, and idiopathic MCD (iMCD, also called HHV-8-negative MCD). Most patients with iMCD present with a mild phenotype designated as not otherwise specified (iMCD-NOS) [2]. Less frequently, patients present with a severe form of iMCD characterized by thrombocytopenia, anasarca, pleural effusions, fevers, reticulin fibrosis or renal dysfunction, and organomegaly (iMCD-TAFRO) [3, 4]. The histopathological subtypes of iMCD are determined based on excisional lymph node biopsy findings, namely the presence of regressed or hyperplastic germinal centers (GCs), follicular dendritic cell (FDC) prominence, vascularity, and plasmacytosis. As part of consensus diagnostic criteria, an expert panel has defined a histopathologic presentation spectrum for iMCD, which ranges from hyaline vascular/hypervascular (characterized by regressed GCs, FDC prominence, and hypervascularity) to plasma cell/plasmacytic (with hyperplastic GCs, and prominent interfollicular plasmocytosis), with mixed subtypes that display a combination of both [5, 6].

While the etiology of iMCD is not fully understood, interleukin (IL)-6 has been identified as the main pathological driver of the disease [1]. This is supported by the positive outcome of anti-IL-6 therapies in clinical trials of patients with iMCD, including the only prospective randomized trial of siltuximab in iMCD [7–9]. The exact event that leads to dysregulated IL-6 in iMCD remains unknown. Underlying autoimmune/autoinflammatory, neoplastic, and pathogenic causes have been hypothesized; a recent study suggests a viral cause is unlikely [10, 11]. It is important to note that IL-6 is not elevated in all patients with iMCD, and other cytokines may be involved [1]. For instance, patients with iMCD-TAFRO have a different cytokine profile, with mildly elevated IL-6 and increased vascular endothelial growth factor (VEGF), as well as highly vascularized lymph nodes. For this reason, the pathology is often referred to as hypervascular rather than hyaline vascular [1]. Overall, patients with iMCD-TAFRO have a poorer prognosis, and some may develop life-threatening cytokine storm with organ failure or even death [12].

Idiopathic MCD is a rare disease with an estimated annual incidence of 3.4 and prevalence of 6.9 cases per million [13]. These estimates have been historically variable because disease-specific International Classification of Diseases (ICD) codes and harmonized diagnostic criteria were only introduced in 2016 and 2017, respectively [5, 6]. These criteria are also referenced in the NCCN Clinical Practice Guidelines in Oncology (NCCN Guidelines) [14]. According to these guidelines (Table 1), a diagnosis of iMCD requires characteristic lymph node histopathology and multicentric lymphadenopathy (major criteria) and ≥ 2 minor criteria (including ≥ 1 laboratory criterion). It is also required to exclude diseases that can mimic iMCD (infections, autoimmune diseases, and malignant/lymphoproliferative disorders). For iMCD-TAFRO, diagnosis requires characteristic lymph node histopathology negative for HHV-8, presence of 4 clinical criteria, and ≥ 1 additional criteria (Table 2) [15].

The IL-6 antagonist siltuximab is a preferred treatment option for iMCD [14, 16]. Siltuximab is also the only FDA-approved treatment for iMCD [1]. It is given as an 11 mg/kg intravenous (IV) infusion every 3 weeks [7, 16]. In addition, the consensus guidelines suggest giving patients diagnosed with severe iMCD weekly siltuximab infusions for 4 weeks [16]. For patients who respond to treatment, siltuximab infusions should be continued indefinitely every 3 weeks with tapering of corticosteroids. Severe iMCD requires the presence of ≥ 2 severe criteria listed in Table 1 [1]. If siltuximab is not available or is not effective/well tolerated, alternatives supported by lower-level evidence in consensus guidelines include tocilizumab (an IL-6 receptor antagonist), rituximab, corticosteroids (as adjunctive therapy to anti-IL-6 agents or rituximab), and/or chemotherapy [16]. Corticosteroid monotherapy is not recommended.

The diagnosis and treatment of iMCD remains challenging because its signs and symptoms are highly variable and often overlap with other diseases. There are no biomarkers specific for iMCD, and diagnosis requires a multidisciplinary team including hematologists, pathologists/hematopathologists, and other specialists. In this report, we present 5 cases that highlight the heterogeneity of this hematological disorder, including 1 case of TAFRO with elusive iMCD. Our aim is to increase awareness for this uncommon disorder and discuss the role of siltuximab in treatment of iMCD.

## 2. Case 1

**Figure 1:**
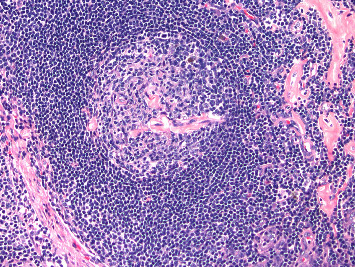
Histopathology results after excisional lymph node biopsy (hematoxylin and eosin stain, Patient 1), showing mixed hyaline and plasma cell type CD: hyaline vascular focus, showing atretic germinal follicle with hyalinized penetrating vessel and concentric mantle zone, and relatively increased adjacent endothelial vessels.

A 49-year-old male with history of hypertension and diabetes mellitus presented with growing pain in the right groin over several months. He denied any constitutional symptoms, peripheral neuropathy, or skin changes. Further laboratory work-up showed hypoalbuminemia (3.0 g/dL, reference range: 3.5–5.5 g/dL) and normal hemoglobin, white blood cells, and platelets along with normal hepatic and kidney functions. Infectious work-up was negative for human immunodeficiency virus (HIV), HHV-8, and Epstein Barr virus (EBV). Further testing showed normal IL-6 levels (reference range < 5 pg/mL) with elevated VEGF (598 pg/mL, reference range: 9–86 pg/mL) and C-reactive protein (CRP, 33.9 mg/mL, reference range: ≤ 3.0 mg/L). No plasma cell dyscrasias were detected on laboratory studies of serum protein electrophoresis with immunofixation (SPEP with IFE) and kappa/lambda ratio (K/L ratio). Computerized tomography (CT) of chest, abdomen, and pelvis showed a right-sided inguinal lymph node measuring 2.3 × 3.0 cm along with other enlarged bilateral external iliac lymph nodes. No hepatosplenomegaly was reported. Positron emission tomography (PET) scan showed similar enlarged lymph nodes in the inguinal regions and external iliac chain with activity of 2.9 maximum standardized uptake value (SUV max). Surgical excision of the lymph node was performed, and pathology showed mixed hyaline vascular and plasma cell types with associated fibrosis confirming MCD (Figure 1). The patient was found to have nonsevere iMCD-NOS and was started on siltuximab 11 mg/kg every 3 weeks. After 3 infusion cycles, the patient experienced complete resolution of groin pain and discomfort, and partial lymph node response (50% reduction in lymph node size) upon repeated scans. We are unable to follow his CRP or the lymph node size as the patient was lost to follow up from our clinic.

## 3. Case 2

**Figure 2:**
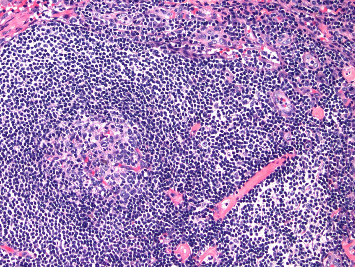
Histopathology results after core needle lymph node biopsy (Patient 2). Hyaline vascular pathology, showing atretic follicle with a penetrating vessel with background increased hyalinized endothelial vessels.

A 35-year-old male with no significant past medical history presented with new onset left upper quadrant abdominal pain ongoing for 2 weeks. The patient admitted to having night sweats and dyspnea that coincided with his abdominal pain. The patient denied any weight loss, easy bruising, or peripheral neuropathy symptoms. Laboratory work-up was normal without any cytopenia. IL-6 levels were normal at 2.8 pg/mL, with normal VEGF (< 31 pg/mL) and CRP (2.1 mg/dL), but erythrocyte sedimentation rate (ESR) was elevated (20 mm, reference range 0–15 mm). Imaging CT scans of the abdomen and pelvis showed multiple enlarged retroperitoneal lymph nodes, most prominent around the pancreas. Hepatosplenomegaly was also reported on abdominal CT. A bone marrow biopsy was unremarkable for any pathology. A retroperitoneal lymph node biopsy was performed by fine needle aspiration (FNA), with pathology results consistent with hyaline variant CD and viral screening negative for HHV-8. IgG4 and IgG stains were negative, excluding IgG4 disease. The patient failed to meet criteria for iMCD-TAFRO or POEMS-iMCD and was diagnosed with iMCD-NOS. Given his dyspnea on exertion and debilitating abdominal pain, the patient was started on prednisone 1 mg/kg, which improved his abdominal pain but was reluctant to continue treatment due to weight gain. The patient was then started on siltuximab 11 mg/kg (IV) every 3 weeks. Abdominal pain persisted after a total of 6 cycles of siltuximab. Repeat scans showed stable disease with no changes in the size of lymph nodes. A repeat core needle lymph node biopsy was pursued to rule out any B-cell lymphoproliferative disorder (histopathology image not available for this patient). The pathology results confirmed hyaline vascular variant CD (Figure 2), and there was no evidence of B- or T-cell lymphoma. A bone marrow biopsy was also repeated, and the results were unremarkable. There was no evidence of monoclonal gammopathy detected on SPEP with IFE and K/L ratio. Given the lack of lymph node response and IL-6 elevation on repeat test (4700 pg/mL), the patient was started on rituximab 375 mg/m^2^ weekly for 4 doses. After limited improvement in his abdominal pain symptoms, the decision was made to restart siltuximab 11 mg/kg (IV) every 3 weeks. Upon retreatment, the patient showed complete response with resolution of lymphadenopathy and continues to tolerate the infusions (every 3 weeks) to date. His CRP continues to be within normal limit.

## 4. Case 3

**Figure 3:**
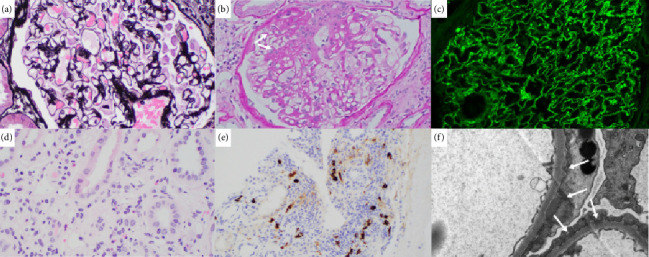
Histopathology results after kidney biopsy (Patient 3): (a) normal appearing glomerulus without obvious spikes or holes along capillary loops (Jones silver stain; 400X magnification), (b) glomerulus with segmental sclerosis near the hilum (see arrows; periodic-acid Schiff, 200X magnification), (c) granular IgG staining along capillary loops and within mesangial regions (immunofluorescence; 400X magnification), (d) mixed interstitial inflammation and edema which includes several eosinophils and plasma cells as well as foci of tubulitis (hematoxylin and eosin; 400X magnification), (e) IgG-4 positive plasma cells with foci of up to 9 positive cells per high-power field (overall IgG4/IgG < 40%; 200X magnification), (f) electron micrograph showing several superficial subepithelial deposits with minimal basement membrane remodeling (see arrows; 110,00X magnification). IgG = immunoglobulin G.

**Figure 4:**
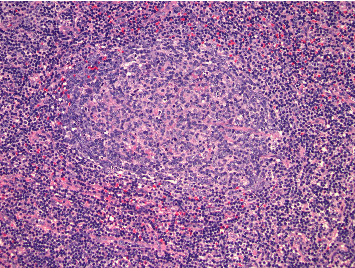
Histopathology results after excisional lymph node biopsy (hematoxylin and eosin stain, Patient 3), showing hyaline vascular pathology.

A 49-year-old female patient with past medical history of asthma and hypertension presented with 2 weeks of painful lumps around her neck. She also experienced fevers, chills, and night sweats. Laboratory work-up was mostly notable for low hemoglobin (9.7 g/dL, reference range: 12–16 g/dL) and low platelets (122 × 10^9^/L, reference range: 150–400 × 10^9^/L). Further testing showed high EBV quantitative polymerase chain reaction (qPCR, 43,995 copies/mL; reference range: not detected), and elevated IL-6 (7 pg/mL), VEGF (860 pg/mL), and CRP (60.3 mg/mL). The patient was also found to be in acute renal failure with reported estimated glomerular filtration rate (eGFR) of 14 mL/min (normal range > 90 mL/min). A kidney biopsy was pursued (Figure 3), which showed membranous nephropathy with segmental sclerosis with acute interstitial nephritis and was negative for Congo red staining. IgG4 and IgG were positive on kidney biopsy however the IgG4/IgG ratio (< 40%) did not support of IgG4 disease. Monoclonal gammopathy was ruled out after SPEP with IFE and K/L ratio. Imaging with CT showed bilateral lymphadenopathy along with mild hepatosplenomegaly. An excisional biopsy of left cervical lymph node showed reactive lymphadenopathy with slightly increased vascularization (HHV-8 negative, Figure 4) and was negative for EBV. Given the overall clinical picture with negative EBV and IgG4/IgG < 40%, both EBV-associated neoplasm and IgG4 disease were excluded, and the patient was diagnosed with iMCD. Given the thrombocytopenia, fever, hepatosplenomegaly, and elevated CRP, TAFRO was considered, but the patient did not meet all clinical criteria for iMCD-TAFRO (patient did not have anasarca). Given the worsening anemia (hemoglobin dropped to 7.8 g/dL), ECOG-PS of 2, and eGFR < 30 mL/min, the patient was diagnosed with severe iMCD-NOS and started on prednisone, then siltuximab in the outpatient setting. The patient received 4 cycles of weekly siltuximab 11 mg/kg and then switched to the same dose every 3 weeks. Concomitant rituximab 375 mg/m^2^ was administered in 4 weekly cycles for EBV eradication, with repeat EBV DNA not detected on qPCR. The patient achieved complete remission with complete resolution of lymphadenopathy on repeat scan. Cell counts and CRP levels normalized after 6 weeks of siltuximab, and the patient continues to tolerate treatment (every 3 weeks).

## 5. Case 4

A 38-year-old female with hypertension, obesity, and seizure disorder presented with diffuse swelling and dyspnea. The patient was found to have ascites, mesenteric edema, pleural effusions, and pericardial effusions on the CT scan at a different hospital. On presentation, the patient had low hemoglobin (8 g/dL) and platelet count (56 × 10^9^/L) along with elevated IL-6 (48,000 pg/mL), VEGF (1300 pg/mL), and CRP (109 mg/L). Bone marrow and core needle lymph node biopsies were performed in the outside facility (histopathology images not available), and the patient received a diagnosis of HHV-8-negative MCD. After the patient was given 1 cycle of rituximab, she was transferred to our hospital given her worsening cytopenia. The patient had thrombocytopenia, anasarca, organomegaly, reticulin fibrosis on bone marrow biopsy, and elevated alkaline phosphatase (ALP, 132 U/L, reference range: 25–100 U/L), meeting the criteria for severe iMCD-TAFRO. She was started on weekly siltuximab 11 mg/kg (IV). After receiving 2 doses, the patient developed posterior reversible encephalopathy syndrome (PRES). Siltuximab was held, and a literature search for any siltuximab-related adverse effect was done. While awaiting decision to continue, the patient received 1 dose of cyclophosphamide, vincristine, and prednisone (CVP). Upon investigation, the adverse event of PRES was determined to be unrelated to siltuximab, and treatment was restarted at the same dose but titrated down to every 3 weeks. Treatment has been well tolerated to date without any adverse events. The patient's condition improved with complete resolution of lymphadenopathy, improvement of dyspnea, normalization of CRP (0.3 mg/dL), and resolution of pleural effusion and ascites.

## 6. Case 5

**Figure 5:**
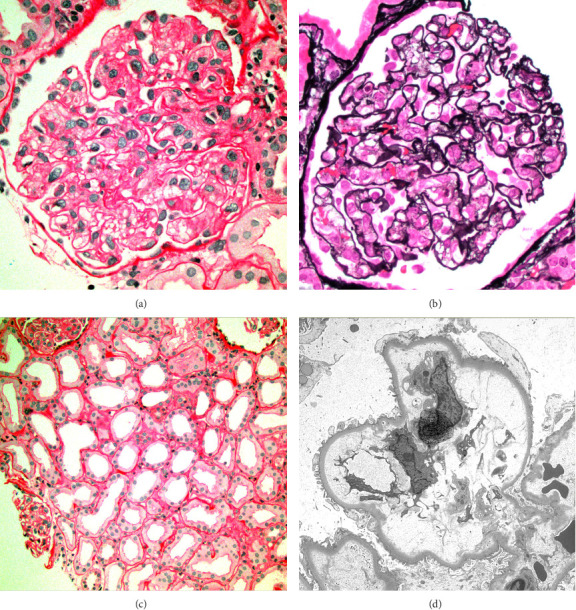
Histopathology results after kidney biopsy (Patient 5): glomeruli with global endothelial cell swelling and mesangiolysis: (a) periodic acid Schiff stain, (b) Jones silver stain; original magnification 400x, (c) diffuse acute tubular injury (periodic acid Schiff stain; original magnification 400x), and (d) electron micrograph demonstrating endothelial cell detachment from basement membranes with interposition of marked flocculent material and mesangiolysis (original magnification 3200x).

A 48-year-old male with bipolar disorder presented with abdominal pain, constipation, and ascites. He was admitted for work-up for ascites and was later found to have generalized anasarca. The patient developed acute renal failure and was started on high-dose steroids for 3 days. His renal function did not improve, and the patient required hemodialysis. Autoimmune work-up was negative for cryoglobulins, lupus nephritis, or other vasculitis. A kidney biopsy showed thrombotic microangiopathy (TMA) with diffuse tubular injury and no evidence of crescents or vasculitis (Figure 5). Laboratory work-up showed low hemoglobin (9.8 g/dL) and platelet count (53 × 10^9^/L). Thrombotic thrombocytopenic purpura (TTP) was ruled out with normal ADAMTS13 activity at 63% (reference range: > 60%). Given the initial suspicion of microangiopathic hemolytic anemia, the patient received 2 doses of eculizumab for atypical hemolytic uremic syndrome (aHUS), but the treatment was eventually stopped after genetic test came back negative. Viral work-up came back negative for HIV and hepatitis but was positive for EBV (qPCR: 4743 copies/mL, reference range: not detected). Given the cytopenia, elevated ferritin (> 1000 ng/mL (reference range: 21.8–274.7 ng/mL), elevated IL-2 (> 4000 pg/mL, reference range: 175.3–858.2 pg/mL), low natural killer cell activity, splenomegaly, and hypertriglyceridemia, the patient met 6 of 8 criteria for hemophagocytic lymphohistiocytosis (HLH) [17]. Bone marrow biopsy showed hypercellular marrow with grade 2-3 reticulin fibrosis but no hemophagocytosis. During the hospitalization, the patient contracted COVID-19 pneumonia which precluded him from getting etoposide for HLH treatment. However, anakinra and dexamethasone were administered for 2 weeks per HLH protocol, along with rituximab (375 mg/m^2^ weekly) for one week for EBV viremia eradication. Another parallel diagnosis considered was TAFRO syndrome with or without iMCD, given thrombocytopenia, anasarca, fevers, reticulin fibrosis, and organomegaly. Lymphadenopathy was absent on repeated imaging, which was thought to be due to 3 days of prednisone on admission and 2 weeks of dexamethasone on HLH protocol. A diagnosis of elusive iMCD was supported by elevated IL-6 (217 pg/mL), VEGF (394 pg/mL), and CRP (216 mg/L), as well as low albumin (1.8 g/dL) and elevated ALP (233 U/L; reference range 46–116 U/L). After 2 weekly doses of siltuximab 11 mg/kg (IV) at our center, there was no significant improvement in cytopenia, but CRP started to trend downward (3.6 mg/mL at discharge). The patient was then transferred to an outside facility for a second opinion, where siltuximab and steroids were held due to suspected COVID-19 sequalae leading to a cytokine storm. The clinical condition was further complicated by fungemia and cytomegalovirus (CMV) viremia, which was determined to be related to siltuximab-induced severe immunosuppression by the transfer institution. It was not until 4 months later that the patient presented with another episode of anasarca and was found to have diffuse lymphadenopathy and elevated CRP (145 mg/L). Core axillary lymph node biopsy showed hyaline vascular/hyper vascular changes, including lymphoid follicles with regressed GCs, and interfollicular polytypic plasmacytosis consistent with CD (negative for HHV-8 and EBV, histopathology image not available). Diagnosis of TAFRO with elusive iMCD was reinstated and siltuximab 11 mg/kg (IV) was restarted weekly for 4 weeks, then every 3 weeks, with concomitant high-dose steroids. The patient showed significant clinical improvement in cytopenia and partial response of lymph nodes on repeat scans. To our knowledge, the patient remains on siltuximab (every 3 weeks) in an outside institution.

## 7. Discussion

We reviewed 5 cases of CD at our institution that represent a wide spectrum of clinical presentations (ranging from constitutional symptoms, elevated inflammatory markers, and lymphadenopathy, to hepatosplenomegaly, anasarca, cytopenia, and acute renal failure; Table 3) and their responses to treatment with siltuximab. Even though CD is a rare and heterogeneous disorder, it should always be considered in the differential of patients presenting with lymphadenopathy. Interestingly, our cases were all multicentric instead of unicentric which is different from the split of approximately 75% UCD and 25% MCD reported in a 2014 epidemiological study [18]. Coincidentally, all 5 of our patients were HHV-8 negative, which is less common than the well-known HHV-8-associated subtype where the virus is the main etiological driver of pathogenesis [10]. Idiopathic MCD-NOS presents as a spectrum from hypervascular to plasmacytic (or mixed) and iMCD-TAFRO can include atrophic GCs with expansion of interfollicular zone and few mature plasma cells [6, 19].

The current case series displays the wide range of symptoms and heterogeneous presentation of iMCD, which can mimic other conditions like infections, autoimmune diseases, and malignant/lymphoproliferative disorders. Excluding these conditions is essential to ensure an accurate diagnosis and appropriate treatment [5, 6]. If the histopathology, imaging scans, and presentation are consistent with MCD, additional work-up for active exclusion of iMCD-mimicking conditions may include flow cytometry, morphological analysis, and immunostaining, as well as additional biopsies particularly to rule out lymphoproliferative neoplasms and autoimmune diseases such as systemic lupus erythematosus (SLE), rheumatoid arthritis, autoimmune lymphoproliferative syndrome, and IgG4 disease. In patients who meet the criteria for iMCD-TAFRO, further evaluation to rule out primary myelofibrosis, SLE, HIV, and HLH is warranted. It is important to raise awareness to iMCD and highlight that this condition should be included in the differential whenever patients present with lymphadenopathy, systemic B-symptoms, and/or multiple-organ system dysfunction [6].

We would like to highlight patient 5, who had a rare presentation of TAFRO with elusive iMCD. This patient had no lymphadenopathy on presentation and developed acute renal failure. He received high-dose steroids for a possible autoimmune process and later received dexamethasone for possible HLH. We hypothesize that the steroids may have initially resolved the lymphadenopathy, which developed once steroids were discontinued. The lab values and pathology results from the lymph node biopsy (showing clear regression of GCs) confirmed the diagnosis of severe TAFRO with elusive iMCD. A similar case reported by Williams et al. describes how a patient met the criteria for TAFRO but could not undergo a repeat biopsy safely (due to low platelets) to confirm iMCD [12]. Instead of delaying lifesaving treatment, the authors highlight the importance of recognizing iMCD early on based on laboratory data (elevated ALP, normal immunoglobulin levels, increased CRP, and hypoalbuminemia) and giving siltuximab as soon as possible [12].

Nishimura et al. reviewed different subcategories of TAFRO: (1) TAFRO with lymph node histopathology consistent with iMCD, (2) possible iMCD-TAFRO (no lymph biopsy could be performed, (3) TAFRO without iMCD [15]. While histopathological findings are important for diagnosis and a lymph node biopsy should always be pursued when feasible, it is important to note that TAFRO syndrome does not require histopathological diagnosis. Two fatal cases reported in the literature were due to TAFRO not being recognized as a separate entity [20]. We intend to emphasize this rare presentation of TAFRO with elusive iMCD, which can help physicians with early recognition of symptoms and prevent decompensation of the patients.

We would also like to call out the kidney involvement in patients 3 and 5. Both patients developed acute renal failure, with patient 5 requiring dialysis. Histopathological findings on kidney biopsy ranged from membranous nephropathy with segmental sclerosis and acute interstitial nephritis (patient 3, Figure 2) and TMA with diffuse tubular injury (patient 5, Figure 4). While the association of CD with kidney disease is uncommon [21, 22], several cases of MCD with concurrent glomerular pathologies like TMA have been described [23–26]. Other kidney findings like membranous nephropathy, segmental sclerosis, and interstitial nephritis are rare [27].

Because of the rarity of the disease, there are no prospective trials comparing different treatment modalities for iMCD. The IL-6 antagonist, siltuximab, is the preferred first-line treatment for iMCD per Castleman Disease Collaborative Network (CDCN) treatment guidelines and NCCN Guidelines [14, 16]. A post hoc analysis showed that siltuximab improves the 2-year estimated progression-free survival (PFS) when compared to placebo (91% vs. 37%) [9]. In a long-term safety and efficacy study of 60 patients with iMCD [8], 70% of patients had durable disease control for up to 6 years and 97% had disease control at the last on-study assessment. There were no deaths among patients who received long-term siltuximab in this study. In a systematic review of patients who received siltuximab for MCD, the 5-year survival rate was estimated at 96.4% [28]. Tocilizumab with or without corticosteroids can also be used as first-line therapy if siltuximab is not available [6]. In patients who do not respond to anti-IL-6 therapies, alternatives can include rituximab and/or chemotherapy, proteasome inhibitors (bortezomib) and immune modulators [6].

IL-6 is an acute-phase cytokine and inflammatory marker of iMCD; it induces B-cell proliferation and is hypothesized to play a key role in the pathogenesis of iMCD [1]. However, some patients have normal or only slightly elevated levels of IL-6, and symptoms are known to wax and wane with IL-6 levels. Current guidelines advise against using IL-6 as a diagnostic criterion and normal IL-6 levels should not preclude patients from receiving siltuximab, which is indicated for treating HHV-8 and HIV-negative MCD regardless of IL-6 status [6]. In fact, some patients who do not have elevated IL-6 obtain clinical benefit from siltuximab (as observed in the Phase II clinical trial [29] and patients 1 and 2). In our 5 cases, all patients responded well to siltuximab with complete or partial response. Out of the 4 patients who presented with lymphadenopathy (Patients 1–4), those who were more symptomatic (met ≥ 3 minor diagnostic criteria) had complete response (Table 4). This corroborates the previous observation that more symptomatic patients with laboratory evidence of inflammation are more likely to benefit from siltuximab [6, 30].

**Figure 6:**
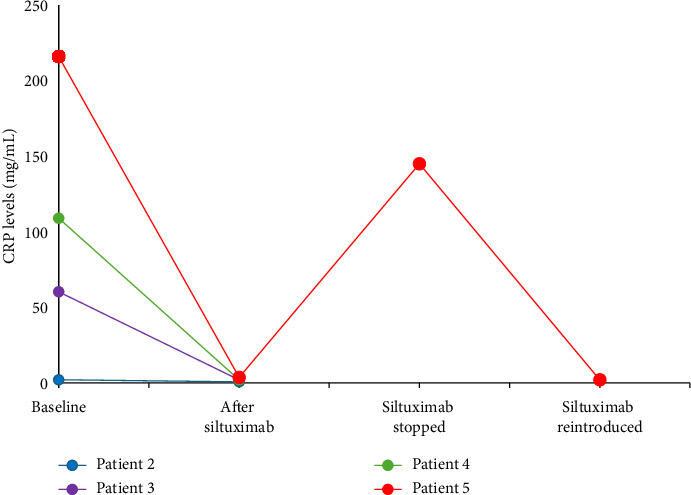
CRP levels after treatment with siltuximab 11 mg/kg. Patient 1 had elevated CRP at baseline but was lost to follow up. CRP = C-reactive protein.

While somatic mutations have been reported in some patients [31–33], iMCD is generally not considered to be a clonal process as the diagnosis is supported by histopathological features of polyclonal plasma cells lacking any somatic mutation or abnormal cytogenetics. No single cell of origin is established as mechanisms usually involve dysregulated production of IL-6 and other cytokines. This triggers a hyperinflammatory state which drives disease pathogenesis, leading to elevation of inflammatory markers such as CRP which is clearly outlined as a laboratory marker in the diagnostic criteria. Siltuximab was effective in normalizing CRP in all patients who had elevated CRP at baseline and ≥ 1 follow-up measurement (Figure 6). In this case series, while only 3 out of 5 patients had elevated CRP and IL-6 at baseline, all 5 responded well to siltuximab. This is consistent with previous data showing that isolated elevation of baseline IL-6 or CRP was not associated with durable tumor and symptomatic response to siltuximab [29]. In iMCD patients treated with IL-6 blockade therapy, spurious increases in serum IL-6 have been reported (as seen in patient 2); in addition, the presence of siltuximab-bound IL-6 may interfere with measurement of unbound bioactive IL-6 [34].

Normalization of CRP, an established biomarker of IL-6 activity [9], is part of an objective response criterion, along with other measurable biochemical markers such as normalization of Hb and improvement of albumin and kidney function [16]. Other indicators of response include reduction in lymph node size and improvement of constitutional symptoms. It should be noted that the time to durable symptomatic response and normalization of laboratory parameters can vary among patients. For instance, Patient 2 of the current case series had persistent abdominal pain and no lymph node response after 6 cycles of siltuximab (without any treatment delays or interruptions) but responded once treatment was restarted. This is in line with a post hoc analysis of patients who responded to siltuximab, where lymph node response increased steadily from 24% at Month 3% to 88% at Month 12, and the median time to achieve durable symptomatic response was 6.9 months [9]. These data show the importance of not prematurely discontinuing treatment.

Importantly, the severity of the disease determines the schedule of siltuximab infusion (every week vs. every 3 weeks, as per treatment guidelines) [16]. A total of 3 patients met the threshold for severe disease (≥ 2 of the following: ECOG-PS ≥ 2, stage IV renal dysfunction, anasarca and/or ascites and/or pleural/pericardial effusions, Hb ≤ 8 g/dL, pulmonary involvement/interstitial pneumonitis with dyspnea), requiring weekly infusion for 4 weeks along with high-dose steroids [16]. In the current case series, Patient 4 had an adverse event of PRES after 2 doses of weekly siltuximab 11 mg/kg. This patient had medically uncontrolled hypertension which is a known risk factor for PRES. Siltuximab was halted and the patient received 1 dose of chemotherapy with CVP. There were no additional episodes of PRES when siltuximab was reintroduced (titrated down to every 3 weeks), and the adverse event was determined not to be related to siltuximab treatment. This is in line with a previous case report of a patient with iMCD-TAFRO syndrome and concurrent Sjögren's syndrome who received siltuximab and steroids [35]. Similarly, all PRES symptoms resolved after treatment with rituximab and CVP. Another AE occurred in Patient 5, who developed fungemia and CMV viremia after receiving siltuximab in the transfer institution. According to an internal investigation, this AE was determined to be related to siltuximab. Siltuximab blocks IL-6 signaling, which might interfere with acute-phase response to pathogens and increase the risk of infection. However, there were no previous reports of siltuximab-associated fungal and CMV infections.

The safety profile of siltuximab has been well characterized in clinical trials and extension studies, and treatment was shown to be well tolerated [7–9]. This safety profile can allow patients to continue the infusions every 3 weeks until treatment failure. In a long-term safety and efficacy study of 60 patients with iMCD, only 2 (3.3%) patients receiving long-term siltuximab had treatment-related serious adverse events (polycythemia and urinary retention) [8]. As part of the open-label safety extension, patients could have their dosing interval extended to every 6 weeks at the investigator's discretion if there was confirmed partial or complete response for > 6 months. Of the 25 patients who transitioned to receiving siltuximab every 6 weeks, 1 patient returned to every 3 weeks dosing due to suspected progressive disease [8]. While this dosing regimen is not FDA-approved, these data suggest that dosing intervals may be extended to 6 weeks in some patients with confirmed partial or complete response for > 6 months, which lessens the need of returning to infusion centers. The decision to extend dosing intervals should be made with caution at the physician's discretion and the patient should be closely monitored for relapse. Currently available guidelines do not address siltuximab discontinuation in patients who had improved symptoms and normalized inflammatory markers. We identified two cases in the literature of iMCD patients with stable disease whom discontinued siltuximab. One patient discontinued therapy after 118 cycles of every 3-week dosing due to pregnancy; this led to recurrence of constitutional symptoms which resolved upon resuming siltuximab [36]. In another case, a patient discontinued treatment after 5 years of every 6-week dosing and experienced disease progression 5 months later; upon resuming siltuximab dosed every 3 weeks, he achieved symptomatic and laboratory remission after two cycles of therapy [37]. Future prospective studies to assess extended interval therapy or therapy cessation upon remission could better inform clinicians regarding disease management.

Our study had limitations due to 2 patients no longer being seen at the clinic (lost to follow-up or no longer living in the area), with no post-baseline CRP measurements for patient 1. Patients 2 and 4 had core needle biopsy, which is not consistent with CDCN or NCCN Guidelines [5, 14]. An excisional biopsy was not feasible for patient 2 due to the abdominal location of the affected lymph node. For patient 4, a core needle biopsy had been performed in the transfer institution and the histopathology results were consistent with iMCD-TAFRO on internal review. Due to the urgency of this case, we determined that an excisional biopsy was not necessary to establish a diagnosis and moved forward with treatment. We were also unable to perform IgG/IgG4 stains to exclude IgG4-related disease on patients 1, 4, and 5 due to either loss to follow-up or biopsies being performed at outside institutions.

## 8. Conclusion

Our single-institution five case review series illustrates different presentations of iMCD and highlights a unique case of TAFRO syndrome with elusive iMCD diagnosis. Early recognition and utilization of diagnostic criteria is important in establishing the diagnosis. Our case series further supports the role of siltuximab as an effective treatment of this disease.

## Figures and Tables

**Table 1:** Consensus diagnostic criteria for iMCD and criteria for severe iMCD [1, 6].

	Major criteria	Minor criteria^a^	
		Clinical	Laboratory
iMCD	• Histopathologic lymph node features consistent with the iMCD spectrum (need grade 2-3 for either regressive GCs or plasmacytosis at minimum) • Multicentric lymphadenopathy (≥ 2 lymph node stations)	• Constitutional symptoms • Skin changes • Enlarged spleen and/or liver, Fluid accumulation • Pulmonary involvement	• Elevated CRP or ESR^b^ • Polyclonal hypergammaglobulinemia • Anemia • Renal dysfunction or proteinuria • Thrombocytosis or thrombocytopenia hypoalbuminemia

Severe iMCD^c^	• ECOG-PS ≥ 2 • Stage IV renal dysfunction • Anasarca and/or ascites and/or pleural/pericardial effusions • Hb ≤ 8 g/dL • Pulmonary involvement/interstitial pneumonitis with dyspnea		

*Note:* Hb = hemoglobin.

Abbreviations: CRP = C-reactive protein, ECOG-PS = Eastern Cooperative Oncology Group Performance Status, ESR = erythrocyte sedimentation rate, GC = germinal center, iMCD = idiopathic multicentric Castleman disease.

^a^At least 1 lab criteria must be met.

^b^Evaluation of CRP is mandatory and tracking CRP levels is highly recommended; however, ESR will be accepted if CRP is not available.

^c^Only 2 of severity criteria must be present for severe disease.

**Table 2:** Diagnostic criteria for iMCD-TAFRO [15].

Histopathology	Clinical criteria^a^	Additional criteria	Supportive criteria^b^
• Lymph node features consistent with the iMCD spectrum. • Multicentric lymphadenopathy (≥ 2 lymph node stations)	• Thrombocytopenia • Anasarca • Fever or hyperinflammatory status (CRP ≥ 2.0 mg/dL) • Organomegaly	• Renal insufficiency/failure • TAFRO-consistent bone marrow, with reticulin fibrosis or megakaryocyte hyperplasia). Supportive clinical criteria (not required for diagnosis) include absence of hypergammaglobulinemia, and ALP without markedly elevated transaminases)	• Hypergammaglobulinemia • High ALP without markedly elevated transaminases

*Note:* ALP = alkaline phosphatase; TAFRO, thrombocytopenia, anasarca, pleural effusions, fevers, reticulin fibrosis or renal dysfunction, and organomegaly.

Abbreviations: CRP = C-reactive protein, ESR = erythrocyte sedimentation rate, GC = germinal center, iMCD, idiopathic multicentric Castleman disease.

^a^All 4 criteria required.

^b^Not required for diagnosis.

**Table 3:** Summary of patient characteristics and clinical course by case.

	Case 1	Case 2	Case 3	Case 4	Case 5
Age (years)	49	35	49	38	48
Sex	Male	Male	Female	Female	Male
Major criteria (need both)					
Histopathology consistent with iMCD spectrum	Yes (mixed)	Yes (HV)	Inconclusive^a^	Yes (NR)	Yes (HV)
Multicentric lymphadenopathy	Yes	Yes	Yes	Yes	Yes
Minor criteria (need ≥ 2)					
Minor criteria—clinical					
Constitutional symptoms	No	Yes	Yes	No	Yes
Enlarged spleen and/or liver	No	Yes	Yes	No	Yes
Fluid accumulation	No	No	No	Yes	Yes
Skin changes	No	No	No	No	No
Pulmonary involvement	No	Yes	No	Yes	No
Minor criteria–laboratory (need ≥ 1)					
Elevated CRP (> 10 mg/mL) or ESR (> 15 mm/h)	↑ CRP (33.9 mg/mL)	↑ ESR (20 mm/h)	↑ CRP (60.3 mg/mL)	↑ CRP (109 mg/mL)	↑ CRP (216 mg/mL)
Anemia (Hb < 12.5 g/dL for M, < 11.5 g/dL for F)	No	No	Yes (9.7 g/dL)	Yes (8 g/dL)	Yes (9.8 g/dL)
Thrombocytopenia (platlet count < 150 k/μL)	No	No	Yes (122 k/μL)	Yes (56 k/μL)	Yes (53 k/μL)
Hypoalbuminemia (< 3.5 g/dL)	Yes (3.0 g/dL)	No	No	No	Yes (1.8 g/dL)
Renal dysfunction (eGFR< 60 mL/min)	No	No	↓ eGRF (14 mL/min)	No	Acute renal failure
Hypergammaglobulinemia (> 1700 mg/dL)	No	No	No	No	No
Additional data					
IL-6 (pg/mL)	< 5	4700	7	48,000	217
VEGF (pg/mL)	598	< 31	860	1300	394
ALP (U/L)	NR	NR	NR	132	394
Reticulin fibrosis of BM	NR	No	NR	Yes	Yes

*Note:* ALP = alkaline phosphatase; F = female; Hb = hemoglobin; IL-6 = interleukin 6; M = male.

Abbreviations: BM = bone marrow, CRP = C-reactive protein, eGRF = estimated glomerular filtration rate, ESR = erythrocyte sedimentation rate, HV = hyaline vascular, iMCD = idiopathic multicentric Castleman disease, NR = not reported, VEGF = vascular endothelial growth factor.

^a^Excisional biopsy of left cervical lymph node showed reactive lymphadenopathy with slightly increased vascularization (HHV-8 negative) and was negative for EBV.

**Table 4:** Treatment outcomes for patients with iMCD in this case series.

	Number of minor criteria	Diagnosis	Treatment response
Patient 1	2	Non-severe iMCD	Partial response
Patient 2	4	Non-severe iMCD	Complete response
Patient 3	6	Severe iMCD	Complete response
Patient 4	5	Severe iMCD-TAFRO	Complete response
Patient 5	8	Severe TAFRO with elusive iMCD	Partial response^a^

*Note:* TAFRO, thrombocytopenia, thrombocytopenia, anasarca, pleural effusions, fevers, reticulin fibrosis or renal dysfunction, and organomegaly.

Abbreviation: iMCD, idiopathic multicentric Castleman disease.

^a^Determined in outside facility.

## Data Availability

The data that support the findings of this study are available from the corresponding author upon reasonable request.
